# RoentMod: a synthetic chest X-ray modification model to identify and correct image interpretation model shortcuts

**DOI:** 10.1038/s41746-026-02497-6

**Published:** 2026-03-06

**Authors:** Lauren H. Cooke, Matthias Jung, Jan M. Brendel, Nora M. Kerkovits, Borek Foldyna, Michael T. Lu, Vineet K. Raghu

**Affiliations:** 1 Cardiovascular Imaging Research Center, Massachusetts General Hospital & Harvard Medical School, Boston, MA USA; 2 Medical Imaging Centre, Semmelweis University, Budapest, Hungary

**Keywords:** Computational biology and bioinformatics, Health care, Mathematics and computing, Medical research

## Abstract

Chest radiographs (CXRs) are among the most common tests in medicine; automated interpretation may reduce radiologists’ workload and expand access. Deep learning multi-task and foundation models have shown strong CXR interpretation performance but are vulnerable to shortcut learning, where spurious correlations drive decision-making. We introduce RoentMod, a counterfactual image editing framework that generates realistic CXRs with user-specified and synthetic pathology while maintaining the original anatomical features. RoentMod combines an open-source medical image generator (RoentGen) with an image-to-image modification model without retraining. In reader studies of RoentMod-produced images, 93% appeared realistic, 89–99% correctly incorporated the specified finding, and all preserved native anatomy comparable to real follow-up CXRs. Using RoentMod, we demonstrate that state-of-the-art multi-task and foundation models frequently exploit off-target pathology as shortcuts, limiting their specificity. Incorporating RoentMod-generated counterfactual images during training mitigated this vulnerability, improving model discrimination across multiple pathologies by 3–19% AUC in internal validation and by 1–11% for 5 out of 6 tested pathologies in external testing. These findings establish RoentMod as a tool to probe and correct shortcut learning in medical AI. By enabling controlled counterfactual interventions, RoentMod enhances the robustness and interpretability of CXR interpretation models and provides a strategy to improve medical imaging models.

## Introduction

Deep learning has achieved remarkable success in CXR interpretation, with performance rivaling experienced radiologists^1–4^. Despite this success, clinical adoption of these tools has remained slow because they (i) generalize poorly to out-of-distribution data^5,6^ and (ii) arrive at decisions differently than humans in ways that may be difficult to explain^7,8^. These problems prevent radiologists from effectively collaborating with models^9,10^, an essential goal for image interpretation systems^11^, and may adversely affect radiologist performance^9^.

One potential driver of both issues is shortcut learning, where models use spurious correlations in their training data to arrive at decisions^12^. In medical imaging, there have been several well-established causes of shortcut learning. First, differences in image appearance and outcome prevalence between institutions or care settings within an institution (e.g., ICU vs. outpatients) can lead to models using “markers” (e.g., marker indicating left side of the patient) for these settings as a “shortcut” for the outcome^5,12,13^. Second, models can accurately estimate demographic and body attributes like age, sex, and size from imaging, leading to biased predictions when outcome prevalence is associated with demographics (e.g., cardiomegaly is more common in older adults)^14–16^. Third, the presence of medical devices (e.g., pacemakers, tubes, and lines) can serve as a surrogate for underlying pathology^17^, are often easier to identify, and have a greater effect on predictions than the pathology itself^1^.

We posit that popular paradigms to train image interpretation models lead to an underappreciated cause of shortcut learning: the use of off-target “tasks” or correlation between outcomes as a surrogate for the outcome itself^18^. Modern models are typically (i) multi-task models trained to predict several derived binary outcomes from text-based reports^1^ or (ii) foundation models trained to generate report text itself^19,20^. Often, these models are trained using large observational cohorts with imaging and text reports. This framework is substantially more efficient than training individual models for each outcome^21^ and has the added benefit of “feature-sharing”^22^ where performance on related tasks improves by simultaneous training. However, multi-task objectives can lead to models encoding image features that are spuriously correlated to multiple outcomes (e.g., supine view indicating sicker patients), as this is often easier than encoding true causal features^18,23^.

**Fig. 1:**
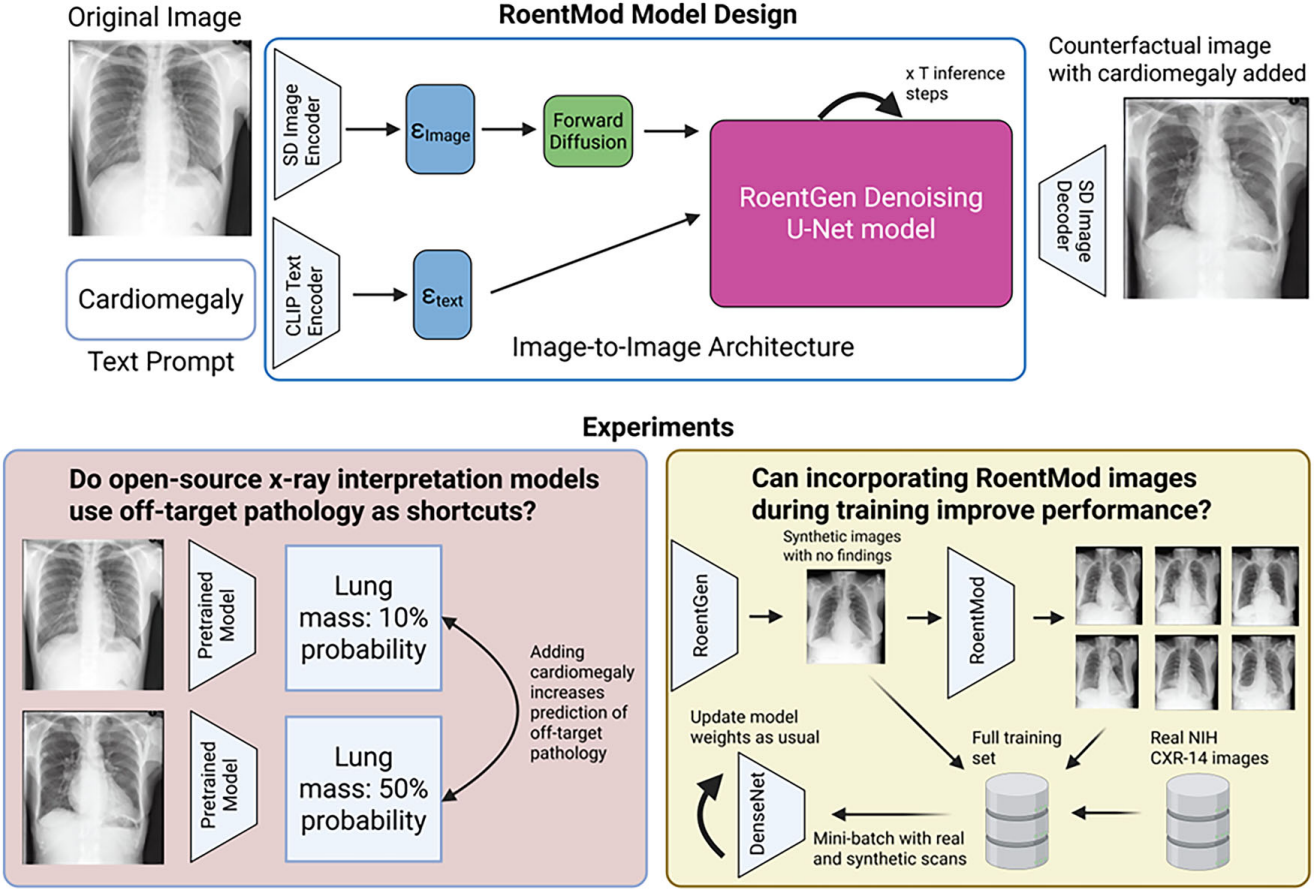
Graphical abstract. RoentMod is a chest X-ray modification model that takes as input a chest radiograph image and a text prompt describing “edits” to be made to the image. RoentMod then outputs a chest X-ray that reflects those edits but maintains the original image’s identity. RoentMod’s internal image-to-image architecture uses publicly available components, including the Stable Diffusion (SD) image encoder, the CLIP text encoder, and the RoentGen denoising U-Net. We then used RoentMod to test whether X-ray interpretation models use off-target pathology as shortcuts and whether incorporating RoentMod synthetic images during training can improve interpretation model performance. SD Stable diffusion, ɛ embedding, CLIP Contrastive language-image pretraining, Created in BioRender. Raghu, V. (2026) https://BioRender.com/boiv0t7.

Current interpretability^24^ and shortcut testing tools^14^ are useful to identify shortcuts but cannot quantify the impact of shortcuts on model performance or mitigate them directly during training. Counterfactual image editing^25^ is a new technique that leverages image generation models to modify an existing image via a text-based prompt. In this way, a pathological finding can be “added” or “removed” from an image (see Fig. 1) while keeping other aspects of the image fixed^26,27^. Current counterfactual CXR editing tools either require expensive hardware^27,28^, lack radiologist expert review and validation^26–28^, or depend on components that are not publicly available^27^.

Here, we introduce RoentMod, a free, open-source, counterfactual image editing tool to add a pathological finding to an existing CXR image. Through experiments on large publicly available datasets and multitask diagnostic models, we make the following contributions:

- We present an accurate counterfactual medical image editing tool (RoentMod) developed by combining a medical image generation model (RoentGen)^38^ with a publicly available image modification model built for natural images (Image-to-Image)^29^ with no additional training of either model.
- We conduct an evaluation study with radiologists, showing that RoentMod accurately modifies real chest radiographs to produce counterfactual radiographs that (a) appear realistic, (b) accurately add a user-specified finding, and (c) largely maintain the other anatomy of the original radiograph.
- We show that using RoentMod to add pathology to a CXR results in increased probability predictions for all tested pathologies (including ones that were not added to the CXR), indicating that state-of-the-art multitask diagnostic models may use shortcut learning.
- We show that a novel training paradigm that combines real scans with their RoentMod-modified counterfactual CXRs in a mini-batch reduces the use of these shortcuts and improves discrimination performance across all pathologies in internal testing on the NIH CXR-14 and MIMIC-CXR Datasets and external testing on PadChest and CheXpert.

## Results

### RoentMod evaluation: RoentMod accurately adds pathology from text prompts while maintaining realism

One radiology resident (4 years of experience) and one board-certified radiologist (8 years of experience) individually assessed a total of 800 (400 each) RoentMod-modified scans blinded to input prompts. Across all tested conditions, RoentMod maintained realistic subject appearance in 745 out of 800 (~93%) synthetic scans. Radiologists observed notable pathology outside of the eight tested in this study in only 20 out of 800 (~4%) scans (Supplementary Fig. 1). When intermixed with real follow-up scans, RoentMod-modified scans maintained a similar level of realism, with realistic subject appearance in 487 out of 504 (~97%) synthetic scans and notable unprompted pathology appearing in only 4 out of 504 (~1%) synthetic scans. Of our eight tested conditions, RoentMod consistently introduced six pathologies when prompted: 92% of cases for cardiomegaly, 97% for edema, 93% for pleural effusion, 89% for pneumonia, 99% for hernia, and 99% for lung mass. When prompted to generate emphysema or lung nodules, RoentMod only added these conditions in 76% and 70% of cases, respectively; however, these results were inconsistent across readers (Supplementary Fig. 2). In general, we found moderate to high inter-rater agreement (Supplementary Table 1) for all findings but edema, emphysema, and real presentations of pneumonia; these findings have well-established low reliability on chest radiographs^30,31^.

### RoentMod evaluation: RoentMod modifies images to add prompted pathology without introducing medically unrelated off-target conditions

Most RoentMod-generated images did not include medically unrelated unprompted conditions (Supplementary Fig. 1). Cardiomegaly frequently appeared when RoentMod was prompted to add edema (92% of cases), which is a reasonable pathophysiology as pulmonary edema is usually related to poor cardiac function and often accompanied by cardiomegaly. Cardiomegaly also appeared frequently in RoentMod images of all other tested conditions (35-50% of cases per prompted pathology). RoentMod also tended to add edema when prompted to add cardiomegaly (46%), emphysema (33%), and lung nodules (50%). Based on these findings, our inter-reader agreement results (Supplementary Table 1), and the relatively lower prevalence of emphysema and nodules when RoentMod was prompted to include them (76 and 70%), we elected to remove nodules and emphysema from the rest of the study.

### RoentMod evaluation: RoentMod preserves the subject’s other anatomy when adding a pathology

To determine whether RoentMod maintains subject identity when adding pathologies, we measured pairwise Fréchet inception distance^32^ (pFID) across the InceptionV3^33^, XResNet^34^, and CLIP^35^ embeddings between baseline real scans with RoentMod synthetic scans from a different person (control score), baseline real scans with RoentMod synthetic scans for the same person (model score), and baseline real scans with real follow-up scans with the added pathology having naturally occurred within two years of the corresponding baseline scan (real score) (Supplementary Table 2). We found that synthetic images were more similar to their baseline real scan than two real CXRs from different individuals. The choice of embedding determined whether synthetic CXRs or real follow-up CXRs were more similar to baseline CXRs from the same person. Using CLIP embeddings, we found that synthetic CXRs were more similar than follow-up CXRs to real images. However, we observed the opposite trend for InceptionV3 embeddings (>10 points per condition) and XResNet embeddings within 5 points for all conditions except 31 points for hernia and 10 for lung masses. We then conducted direct image-level analysis and found that RoentMod-edited images showed pixel intensity changes only in the correct anatomic region for each pathology, and there were no major differences in pixel intensity distributions after RoentMod editing (Supplementary Fig. 3).

### Using RoentMod to add pathology to CXRs results in higher predicted probability of other pathology

**Fig. 2:**
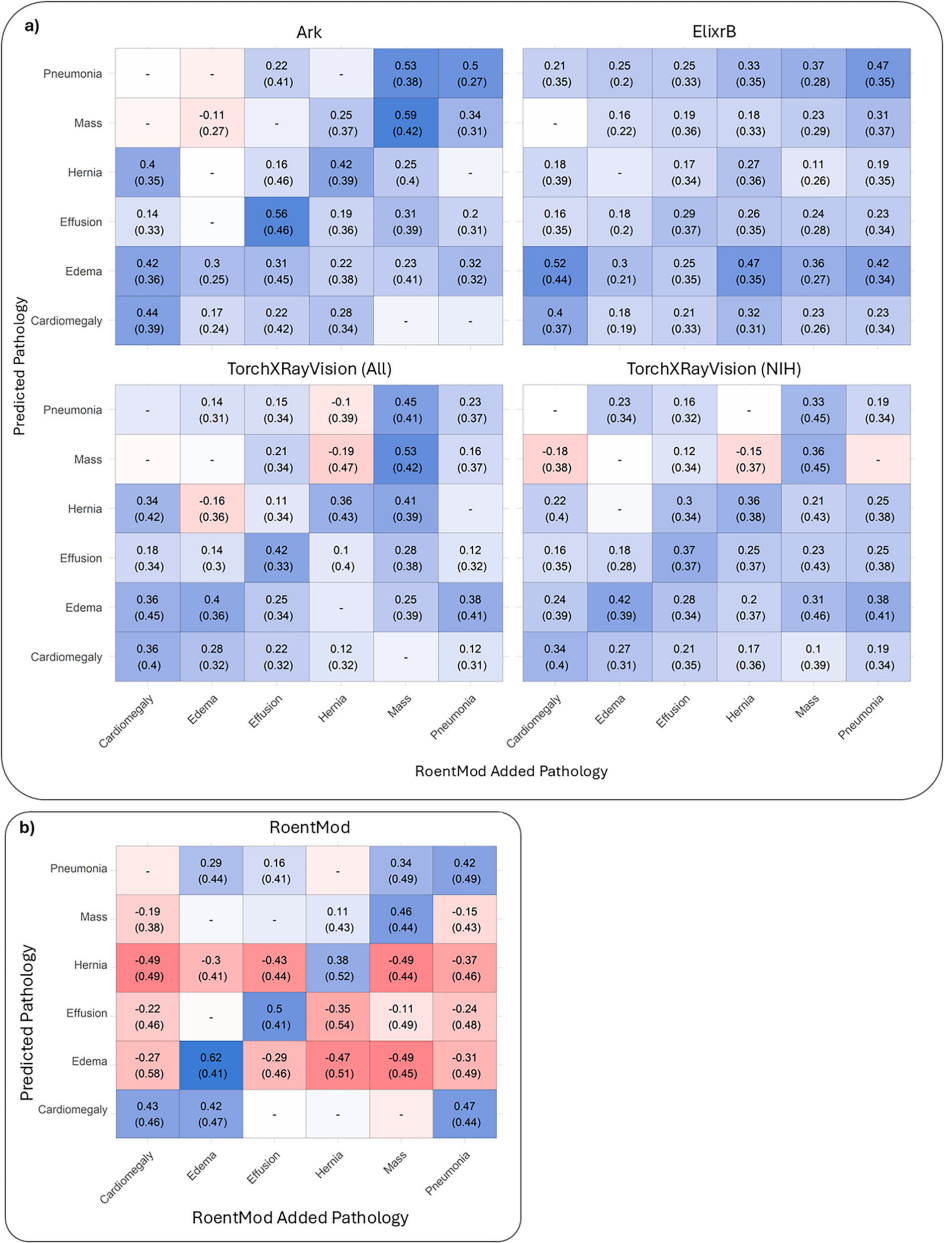
Effect of adding pathologies on predicted probabilities from existing multitask CXR interpretation models. **a** and our RoentMod-trained multitask CXR interpretation model (**b**) on MIMIC-CXR synthetic scans. Blue boxes indicate greater sensitivity to adding pathology. Blank boxes indicate no significant change between baseline and RoentMod-generated counterfactuals. Values reflect the median change in predicted probability percentile after pathology is added to scans with no finding in MIMIC-CXR. So, the value 0.23 (0.34) in (**b**) in the pneumonia row and edema column represents that median pneumonia predictions increased 0.23 percentile, or rank within the distribution, when RoentMod added Edema with an interquartile range of 0.34 percentile. We refer to the TorchXRayVision model trained on all cohorts as TorchXRayVision (All) and the TorchXRayVision model trained on the NIH CXR-14 cohort only as TorchXRayVision (NIH).

We then used RoentMod to “stress-test” existing multitask and foundation X-ray interpretation models (Supplementary Table 3). To do this, we assessed the change in predicted probability between pairs of original images from the MIMIC-CXR cohort without pathological findings and their RoentMod-modified counterparts after adding a pathology (Fig. 2a). We then identified potential shortcuts by assessing the change in predicted probability percentile for all tested pathologies when adding a single pathology using RoentMod.

We found that all tested models likely exhibit shortcut learning, as adding a single off-target pathology changed the diagnostic model’s prediction probabilities for all other tested pathologies (e.g., adding edema changes the prediction of hernia) (Fig. 2a; off-diagonal entries). We verify this behavior using change in saliency across input real and synthetic scans, where we found that TorchXRayVision (NIH) focuses on the added condition and not the area where the predicted condition is likely to be when predicting off-target pathologies (Supplementary Fig. 4). TorchXRayVision (All) exhibited strong changes in predicted probability for all findings when RoentMod added a lung mass, pleural effusion, cardiomegaly, or pulmonary edema. The multi-task model trained only on the NIH cohort had less evidence of shortcut learning based on a change in predicted probability when adding an off-target finding. The ElixrB model demonstrated strong shortcut learning for all pathologies tested, especially when predicting pneumonia or pulmonary edema (percentile change from 58 to 75%). The Ark+ foundation model had the smallest use of shortcut learning, but still used the presence of a lung mass as a shortcut for all findings (percentile increase from 20 to 58%). Both foundation models exhibited a strong tendency to identify pulmonary edema on counterfactual scans regardless of the added condition. We found similar results when using baseline scans from NIH CXR-14 (Supplementary Fig. 5).

### Using RoentMod scans during training reduces reliance on shortcuts and improves generalization

We then trained a multi-task model using both real scans from NIH CXR-14 and RoentMod-generated counterfactual scans to mitigate shortcut learning. We first compared discrimination (area under the ROC curve, or AUC) on held-out NIH CXR-14 (in-distribution), MIMIC-CXR (RoentGen-training cohort), CheXpert (out of distribution), and PadChest (out of distribution) testing sets. We found that incorporating RoentMod-modified scans generally improves diagnostic accuracy beyond a multi-task model trained only on real NIH CXRs (TorchXRayVision (NIH)). We found a particularly large AUC improvement (3–19%) in NIH CXR-14 and MIMIC-CXR (Table 1). Notably, we found that our RoentMod-trained multitask diagnostic model’s in-distribution AUC surpassed zero-shot predictions from foundation models trained on much larger cohorts. We further note that all tested models besides ElixrB were trained on NIH CXR-14 with different train-test splits, so reported AUCs are likely overestimates. In contrast, in out-of-distribution data (PadChest and MIMIC-CXR), we find that models trained on much larger cohorts outperform our RoentMod-trained multitask model.

**Table 1:** Discrimination (AUC) of publicly available and RoentMod-trained multi-task models across diverse cohorts

NIH CXR-14 AUC	Cardiomegaly	Edema	Pleural effusion	Pneumonia	Hernia	Mass
RoentMod (NIH)	**0.95 [0.95,** **0.95]**	**0.901 [0.88,** **0.92]**	**0.909 [0.91,** **0.91]**	**0.746 [0.73,** **0.77]**	**0.922 [0.9,** **0.95]**	**0.854 [0.85,** **0.86]**
TorchXRayVision (NIH)	0.907 [0.9, 0.91]	0.766 [0.73, 0.8]	0.862 [0.86, 0.87]	0.678 [0.66, 0.7]	0.82 [0.79, 0.85]	0.81 [0.8, 0.82]
TorchXRayVision (All)	0.907 [0.9, 0.91]	0.805 [0.78, 0.83]	0.88 [0.88, 0.88]	0.673 [0.65, 0.7]	**0.927 [0.9,** **0.95]**	0.835 [0.83, 0.84]
Ark	**0.939 [0.93, 0.94]**	**0.874 [0.85,** **0.9]**	**0.911 [0.91,** **0.91]**	**0.758 [0.74,** **0.78]**	0.891 [0.87, 0.92]	**0.902 [0.9,** **0.91]**
ElixrB	0.802 [0.79, 0.81]	0.841 [0.82, 0.86]	0.876 [0.87, 0.88]	0.701 [0.68, 0.72]	0.681 [0.65, 0.72]	0.786 [0.78, 0.79]

MIMIC-CXR AUC	Cardiomegaly	Edema	Pleural effusion	Pneumonia	Hernia	Mass
RoentMod (NIH)	**0.809 [0.81,** **0.81]**	**0.76 [0.75,** **0.77]**	**0.881 [0.88,** **0.88]**	**0.541 [0.54,** **0.55]**	**0.881 [0.87,** **0.89]**	0.606 [0.6, 0.61]
TorchXRayVision (NIH)	0.756 [0.75, 0.76]	0.682 [0.67, 0.69]	0.813 [0.81, 0.82]	0.521 [0.52, 0.53]	0.66 [0.64, 0.68]	**0.617 [0.61,** **0.62]**
TorchXRayVision (All)	0.84 [0.84, 0.84]	0.779 [0.77, 0.78]	0.871 [0.87, 0.87]	0.554 [0.55, 0.56]	**0.893 [0.88,** **0.9]**	0.644 [0.64, 0.65]
Ark	**0.859 [0.86,** **0.86]**	**0.829 [0.82,** **0.83]**	**0.929 [0.93,** **0.93]**	**0.61 [0.61,** **0.61]**	0.846 [0.83, 0.86]	**0.74 [0.73,** **0.75]**
ElixrB	0.806 [0.8, 0.81]	0.789 [0.78, 0.79]	0.905 [0.9, 0.91]	0.553 [0.55, 0.56]	0.696 [0.68, 0.71]	0.62 [0.61, 0.63]

CheXpert AUC	Cardiomegaly	Edema	Pleural effusion	Pneumonia	Hernia^a^	Mass
RoentMod (NIH)	**0.798 [0.79,** **0.81]**	**0.762 [0.75,** **0.77]**	**0.851 [0.85,** **0.86]**	**0.549 [0.53,** **0.57]**	NA	**0.578 [0.57,** **0.59]**
TorchXRayVision (NIH)	0.734 [0.72, 0.74]	0.702 [0.69, 0.71]	0.822 [0.82, 0.83]	0.525 [0.51, 0.54]	NA	0.565 [0.55, 0.58]
TorchXRayVision (All)	0.803 [0.79, 0.81]	0.831 [0.82, 0.84]	0.882 [0.88, 0.89]	0.653 [0.64, 0.67]	NA	0.632 [0.62, 0.65]
Ark	**0.869 [0.86,** **0.88]**	**0.899 [0.89,** **0.91]**	**0.927 [0.92,** **0.93]**	**0.748 [0.73,** **0.76]**	NA	**0.721 [0.71,** **0.73]**
ElixrB	0.781 [0.77, 0.79]	0.79 [0.78, 0.8]	0.888 [0.88, 0.89]	0.655 [0.64, 0.67]	NA	0.596 [0.58, 0.61]

PadChest AUC	Cardiomegaly	Edema	Pleural Effusion	Pneumonia	Hernia	Mass
RoentMod (NIH)	**0.907 [0.9,** **0.91]**	**0.796 [0.78,** **0.81]**	**0.847 [0.84,** **0.85]**	**0.675 [0.66,** **0.68]**	**0.898 [0.89,** **0.91]**	0.833 [0.82, 0.85]
TorchXRayVision (NIH)	0.89 [0.89, 0.89]	0.762 [0.74, 0.78]	0.832 [0.83, 0.84]	0.664 [0.65, 0.67]	0.784 [0.77, 0.8]	**0.841 [0.82,** **0.86]**
TorchXRayVision (All)	0.926 [0.92, 0.93]	0.792 [0.77, 0.81]	0.882 [0.88, 0.89]	0.744 [0.73, 0.75]	**0.958 [0.95,** **0.96]**	0.9 [0.89, 0.91]
Ark	**0.927 [0.92,** **0.93]**	**0.869 [0.86,** **0.88]**	**0.939 [0.94,** **0.94]**	**0.877 [0.87,** **0.88]**	0.862 [0.85, 0.87]	**0.932 [0.92,** **0.94]**
ElixrB	0.886 [0.88, 0.89]	0.84 [0.83, 0.85]	0.889 [0.88, 0.89]	0.781 [0.77, 0.79]	0.713 [0.7, 0.72]	0.854 [0.84, 0.87]

We refer to the TorchXRayVision model trained on all cohorts as TorchXRayVision (All) and the TorchXRayVision model trained on the NIH CXR-14 cohort only as TorchXRayVision (NIH).

^a^Hernia was not recorded in the CheXpert database.

The bold values should represent the highest AUC values per column per cohort split into two groups. The first group is the set of models trained on the single NIH cohort (RoentMod (NIH) and TorchXRayVision (NIH)), or the first two rows of each cohort’s table. The second group is the set of models trained on all cohorts (TorchXRayVision (All), Ark, and ElixrB), or the last three rows of each cohort’s table.

We then tested our RoentMod-trained multitask model using the same counterfactual stress test approach (Fig. 2b). We found that our model largely corrects shortcut learning on counterfactual scans for all tested conditions, except the model still overrepresents presence of pneumonia on scans with added lung masses and cardiomegaly on scans with added pulmonary edema when compared to the radiologist-read co-occurrence rates of these pathologies as ground truth. These are reasonable, as pneumonia is common in lung cancer^36^ and the typical presentation of edema is cardiogenic. Of note, due to the counterfactual scans only adding one finding at a time, the model trained using these scans tends to exhibit negative shortcut learning, underpredicting findings when another is present. We found similar results when using NIH CXR-14 baseline scans.

## Discussion

Chest radiographs (CXRs) are among the most common tests in medicine^37^, and accurate, automated interpretation is poised to greatly reduce radiologists’ burden^11,19^. Since many pathologic findings may appear on CXR, multi-task and foundation models are more efficient and can be more accurate than training specialized models for each task. However, they are also more vulnerable to shortcut learning, where models rely on spurious correlations to make decisions. Here, we introduced RoentMod to identify and reduce shortcut learning. RoentMod generates anatomically realistic counterfactual CXRs with synthetic pathology while preserving the original features on the image. Using RoentMod, we show that multi-task interpretation models and foundation models use off-target pathology as shortcuts and that incorporating RoentMod-generated images during training removes this shortcut and can improve interpretation models.

Our major findings are:

- An accurate counterfactual medical image editing tool can be developed by combining a medical image generation tool (RoentGen^38^) with the publicly available image modification tool (image-to-image^29^), with no additional training of either model.
- In user studies with board-certified radiologists, RoentMod accurately modifies real chest radiographs to produce synthetic radiographs that (a) appear realistic 93% of the time, (b) accurately add a user-specified finding 89-99% of the time, and (c) maintain the anatomy of the original radiograph akin to real follow-up scans.
- Many publicly available multi-task and foundation models use off-target pathologic findings as shortcuts, suggesting that these models lack specificity to individual pathology.
- A training paradigm that incorporates RoentMod counterfactual images together with real images corrects this shortcut learning and improves discrimination compared to a model trained on the same real dataset in internal (3–19% AUC improvement) and external testing (improvement on 5/6 pathologies).

Several studies have reported shortcut learning by image interpretation models including CXR models^12–14^. These have largely focused on protected demographic attributes like age, sex, and racial identity or image acquisition/technical parameters^5^. Here, we use RoentMod’s counterfactual image generation to show that both conventional multi-task learning and foundation model training lead to models that use off-target pathologic findings as “shortcuts” (i.e., generally sicker individuals have higher predictions for all pathologic findings). We show that vetting the counterfactual image generation tool through radiologist evaluation and then incorporating counterfactual images during training can mitigate shortcut learning and lead to more generalizable models with minimal training data.

Counterfactual medical image generation is growing in popularity with several tools recently made available^26–28^. RoentMod distinguishes itself through its ease of use and computational efficiency. RoentMod is a combination of publicly available image modification and CXR generation models and required no additional training to generate anatomically realistic counterfactual CXRs. These CXRs are generated in <2 s/image on consumer GPUs (NVIDIA RTX 4090) and <30 s/image on a standard CPU (12th Gen Intel Core i5-12500T with 16 GB of RAM). Additionally, we conducted a thorough evaluation of RoentMod using both radiologist and in silico studies and found that RoentMod generates anatomically realistic radiographs that adhere to a user-specified text prompt and preserve the subject’s anatomy.

While our study focused on mitigating shortcuts based on off-target pathology in CXR image interpretation models, RoentMod and other counterfactual medical image generation tools could be used in numerous other medical imaging applications. Examples include fairness evaluations to ensure that demographic changes do not result in substantially different interpretation performance, and stress-testing and fine-tuning segmentation models to ensure that anatomic segmentation is unbiased based on the presence/absence of pathology. Currently, a major focus of AI research is on foundation models and automated report generation from medical images^19,21,39^. Here, we show that the zero-shot classification performance of a foundation model suffers from the same shortcuts as multi-task interpretation models, suggesting that counterfactual images may improve report generation tools as well. This is especially important in medical imaging, where achieving the massive scale of other foundation models (e.g., large language models) is impractical due to data regulation, patient privacy, and limited ability to acquire additional data.

Limitations of our study should be considered. In this work, we focused on synthetically modifying CXRs to add eight common pathologies; however, a major benefit of foundation models is that they can simultaneously identify many pathologies. As with RoentGen^38^, RoentMod requires clearly defined pathologies (e.g., “multiple pulmonary nodules” vs. “nodule”) with short context in user text prompts, and here we only robustly test eight common pathologies available in the MIMIC-CXR dataset. Incorporating RoentMod images during training improved chest radiograph interpretation performance across several external cohorts; however, RoentMod was only trained on CXRs from MIMIC-CXR, a Boston-based cohort. Fine-tuning RoentMod on more diverse datasets may improve the diversity and reduce institutional bias of synthetically generated images. Here, we focused on CXR, but we expect that interpretation models for other imaging modalities will likely suffer from similar shortcuts; this will be tested in future work. We also observed that co-occurrence rates between some pathologies were higher than expected in synthetic scans (e.g., cardiomegaly often appeared regardless of the user prompt). Additional techniques (e.g., structural causal models, region-specific editing) or other image-to-image model architectures could be used to improve RoentMod and further mitigate these correlations in future work. Our model trained using RoentMod counterfactual images exhibited a slight “reverse” shortcut learning since we generated images with exactly one pathology. Generating multiple pathologies on a single image or recalibration during training may be necessary to prevent this and may result in improved generalization performance. We also note that our RoentMod-trained diagnostic model only had exposure to real NIH CXR-14 scans. Although performant on this limited dataset in comparison to its multitask diagnostic model counterparts, we hypothesize that a multitask diagnostic model trained on more examples across more cohorts with this RoentMod-supplemented training paradigm could potentially perform at or better than these larger models. We used an in-silico analysis based on pFID to ensure subject-identity preservation; however, these results were inconsistent across embedding models. Future work should explore better metrics to evaluate similarity between real and edited images. Lastly, subtle changes may be present in counterfactually generated CXRs that are not visible to human readers, affecting our ability to accurately perform shortcut testing.

In summary, we introduced RoentMod, a counterfactual CXR image generation tool to identify and mitigate shortcut learning in CXR interpretation models. RoentMod generates realistic counterfactual images that adhere to a text prompt without changing unrelated anatomy. Using RoentMod, we find that multi-task and foundation models use the presence of any pathology as a shortcut to interpret CXRs. Finally, using real and synthetic CXRs during training removes this shortcut and improves the generalization performance of multi-task interpretation models.

## Methods

This work relies solely on publicly available datasets and models. Readers who have completed the necessary access requirements can use all datasets in the study. All resources produced in this study, including the RoentMod synthetic scan modification tool, a sample dataset of synthetic scans, all statistical analysis code, and radiologist reads of synthetic scans, are publicly available to use for further research.

### Datasets

We collected and used three de-identified chest X-ray (CXR) datasets with associated pathology labels. All pathology co-occurrence rates and pathology frequency by cohort are given in Supplementary Fig. 6 and Supplementary Table 4.

#### Datasets: National Institutes of Health (NIH) chest X-ray 14^40^

This cohort consists of 112,120 inpatient frontal radiographs from 30,805 patients collected between 1992 and 2015 at the NIH Clinical Center in Bethesda, MD^40^. We only used the posterior-anterior (PA) scans taken on patients aged 18 or older, for a final cohort of 64,628 scans over 27,713 patients (mean age 47.8 $\pm \,$15.0 years, 47% female). For each image fourteen text-mined disease labels were extracted from the corresponding scan’s radiology report into a database: atelectasis, consolidation, infiltration, pneumothorax, edema, emphysema, fibrosis, pleural effusion, pneumonia, pleural thickening, cardiomegaly, nodule, mass, and hernia. Entries without any findings from this list were labeled no finding.

#### Datasets: MIMIC-CXR^41^

This cohort consists of 227,835 CXR imaging studies (scan and radiology report) from 65,379 patients collected between 2011 and 2016 at the Beth Israel Deaconess Medical Center in Boston, MA. We limit our work to frontal scans and patients aged 18 or older, for a final cohort of 94,067 scans over 44,642 patients (mean age 56.1 $\pm \,$19.0 years, 53% female). We then used the labels that MIMIC-CXR produced on their dataset with the CheXpert labeler^42^, where each image contains fourteen text-mined pathology presence labels from the corresponding scan’s radiology report: no finding, atelectasis, consolidation, pneumothorax, edema, pleural effusion, pneumonia, pleural other, cardiomegaly, lung lesion, lung opacity, enlarged cardio mediastinum, fracture, and support devices. Any values labeled as uncertain were treated as negative labels for the presence of that condition.

#### Datasets: CheXpert^42^

This cohort consists of 224,316 inpatient and outpatient radiographs from 65,240 patients collected between 2002 and 2017 at Stanford Hospital in Palo Alto, CA. We only used PA radiographs for patients 18 or older, for a final cohort of 29,453 scans over 20,574 patients (mean age 57.1 ± 17.7 years, 38% female). Each image contains fourteen text-mined pathology presence labels from the corresponding scan’s radiology report: no finding, atelectasis, consolidation, pneumothorax, edema, pleural effusion, pneumonia, pleural other, cardiomegaly, lung lesion, lung opacity, enlarged cardio mediastinum, fracture, and support devices. Any values labeled as uncertain were treated as negative labels for the presence of that condition.

#### Datasets: PadChest^43^

This cohort consists of 168,861 CXR imaging studies (scan and radiology report) from 67,625 patients collected between 2009 and 2017 at the *Hospital Universitario de San Juan* in Alicante, Spain. We limit our work to frontal scans and patients aged 18 or older, for a final cohort of 88,109 scans over 59,085 patients (mean age 58.6 $\pm \,$17.4 years, 52% female). We used labels extracted from the radiology reports as described by the PadChest curators^43^.

### Related work

Shortcut learning in medical image interpretation is the tendency for deep learning models to use spurious correlations in training data instead of the actual presentation of the pathology to make decisions. Most techniques to identify shortcut learning have focused on model interpretability more broadly. These fall into three main categories: heatmap-based techniques, shortcut testing, and counterfactual image editing.

Heatmap-based approaches (e.g., saliency maps^44^, Grad-CAM^45^, and its variants)^46,47^ aim to find areas in the image that most strongly affect a model’s output. These techniques assign an importance value to each pixel or region of an image to produce a heat map of influence for a model’s prediction^48^. These importance values typically correspond to the gradient of the prediction with respect to the pixel value (i.e., how much the output would change if the pixel value changed). This approach has been used to successfully identify visually apparent shortcuts in multitask medical imaging diagnostic models^5^; however, saliency maps require experts to manually review large sets of maps to identify shortcuts^13^. More concerningly, recent studies have found saliency maps can remain nearly identical after randomizing model weights, suggesting that saliency maps capture dataset-specific patterns (e.g., edges in an image) rather than a model’s particular decision-making process^24,49,50^.

Shortcut testing techniques aim to directly test whether trained models rely on user-specified shortcuts by investigating models’ latent representations. Recent work has shown that models tend to have reduced prediction depth (layer at which a prediction is finalized) when using an obvious shortcut^51^. Others have relied on the assumption that spatial features are less likely to be shortcuts than pixel intensity-based representations^52^ to identify shortcuts and improve fairness^12,53^. Another technique, ShorT^14^, assumes that shortcuts will reduce fairness metrics across a hypothesized shortcut attribute and tests whether model-based encoding of the attribute reduces fairness for the real target. For both heatmap-based and direct shortcut testing, it is unclear how to mitigate shortcuts once identified. Image augmentation techniques appear to improve fairness^53,54^ but likely do not prevent all shortcuts.

Counterfactual generation techniques create new input images that remove or alter image features to directly investigate shortcuts. The first set of these techniques (e.g., Gifsplanation, Generative Visual Rationales)^55,56^ use Generative Adversarial Networks (GANs)^57^ and Variational Autoencoders (VAEs)^58^ to produce counterfactual images most similar to the original image but with a different model prediction. While GAN-based methods have successfully found realistic features in models trained to predict heart failure in cardiac MR^59^ and CXR^60^, they also produce lower resolution generations and suffer from unstable training regimes^61^.

Modern approaches use Stable Diffusion’s open-source image generation models^29^ and fine-tuned medical image versions (e.g., RoentGen^38^) to find shortcuts using text-guided, high-quality medical imaging counterfactuals. BioMedJourneys^28^ fine-tuned Stable Diffusion with GPT-4-based instruction fine-tuning to generate image counterfactuals from a baseline image and associated text describing the changes. This approach accurately produces counterfactual images but suffers from hallucinations and a lack of radiologist-based synthetic image evaluation. RadEdit^27^ introduced a diffusion-based generative model to edit chest radiographs using human-drawn image masks to restrict editing to relevant areas while minimizing unrelated artifacts (e.g., introduction of support devices). RadEdit can produce counterfactual images through image editing; however, it is not known whether RadEdit can identify shortcuts or mitigate identified model biases. Further, it is difficult to apply RadEdit at scale because it requires input bounding boxes for each image. PRISM^26^ reconsiders this image editing approach by removing the need for image masks by fine-tuning the Stable Diffusion Text-to-Image^29^ model’s denoising U-Net directly to edit CXRs and permit higher-resolution image generation (512 × 512 pixels). Using PRISM-generated images during training improves classifier performance for four pathologies. However, PRISM can only edit findings documented in CheXpert^42^, and the effectiveness of PRISM’s generated images was evaluated using classifier prediction changes, which may not reflect radiologist interpretation. A set of novel approaches aims to use structural causal models to ensure that counterfactual images avoid spurious correlations during generation^62,63^. While promising, these approaches require an accurate description of the data-generating process, including all confounders, and more work is required to generate high-resolution medical images efficiently.

Building on previous image editing work, RoentMod leverages existing models instead of retraining from scratch. We create a diffusion-based image editor by combining RoentGen’s pretrained U-Net and text encoder weights with pretrained weights from Stable Diffusion’s Image-to-Image model^29^, requiring no additional training. Crucially, we collaborate with board-certified radiologists to validate RoentMod, rather than relying on model-based validation. We can then use generated images not only to reveal shortcut learning in interpretation models but also to incorporate synthetic images during training to mitigate these shortcuts. This work aims to make AI-based medical imaging tools more trustworthy in correcting these shortcuts, taking an important step toward bridging the gap between research and clinical use for these tools.

### Approach overview

**Fig. 3:**
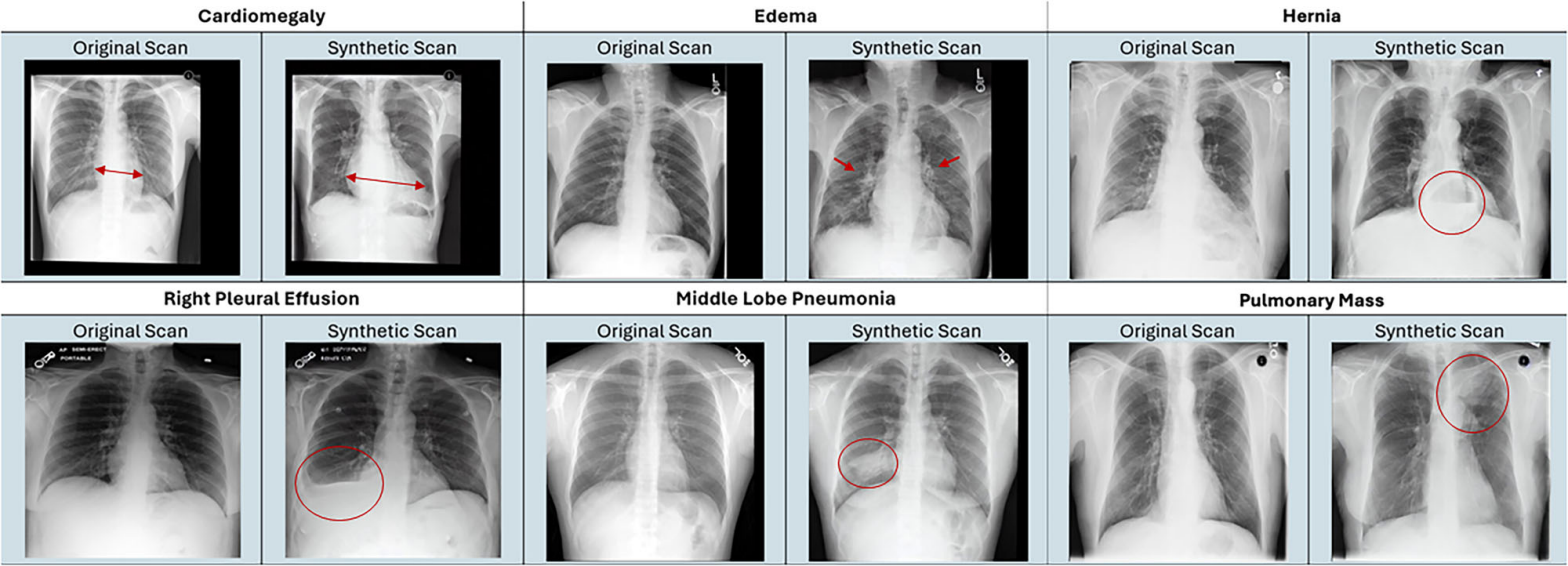
Representative real and counterfactual annotated chest radiographs produced by RoentMod. Each panel represents a disease RoentMod was prompted to introduce. The left image within the panel represents the original scan with no marked findings, while the right image within the panel represents the RoentMod-modified synthetic scan that contains the introduced finding. The red markings represent the locations of each introduced finding, while the red mark on the original scan for cardiomegaly represents a reference smaller heart size that makes the cardiomegaly more apparent in the synthetic scan.

We aimed to develop a generative model to modify CXRs based on a user-specified text prompt. Our model, RoentMod, combines the CXR knowledge encoded in the weights of the RoentGen^38^ CXR generation model with the Stable Diffusion Image-to-Image architecture^29^ for image modification. RoentGen^38^ is the Stable Diffusion text-to-image diffusion model^29^ finetuned on MIMIC-CXR to produce synthetic CXR images that adhere to a user-specified text prompt. We first assessed the quality of RoentMod synthetic scans (Fig. 3) across three dimensions: (1) realism and adherence to the text prompt (e.g., adding a right upper lobe lung mass when prompting “right upper lobe mass”), (2) limited addition of unrelated conditions (e.g., adding hernia when prompting “right upper lobe mass”), (3) subject-identity preservation (maintaining the individual’s anatomy outside of the prompted pathology). Second, we used RoentMod to evaluate how existing CXR interpretation models’ predictions change with the addition of off-target pathology (e.g., adding cardiomegaly when predicting pneumonia) by comparing predictions across pairs of real unmodified scans with “normal” chest X-rays (i.e., no finding noted in the radiology report) and their RoentMod synthetic counterparts after adding pathology. We interrogated four pretrained models in this work: two models from the TorchXRayVision^64^ library (TorchXRayVision trained on all cohorts named TorchXRayVision (All), TorchXRayVision trained on the NIH CXR-14 cohort only named TorchXRayVision(NIH)), the ElixrB CXR foundation model^39^, and the Ark+ CXR foundation model^19^ (Supplementary Table 3). We performed this analysis on sets of seven scans per patient: one original unmodified scan and six RoentMod-modified synthetic versions, where each modified scan contained a preselected pathology. This allowed us to assess how the addition of one pathology affects the model’s predictions for all other pathologies. Finally, we trained our own diagnostic models on synthetic and real scans to attempt to remove reliance on shortcuts.

### RoentMod design: no training required

RoentMod allows a user to input an existing CXR and a text prompt to get a new scan of that individual that reflects changes requested by the prompt (Fig. 1). To build RoentMod, we applied the pretrained weights from RoentGen^38^ to the Stable Diffusion Image-to-Image architecture^29^. We used the finetuned weights for the text encoder and Denoising U-net from RoentGen and the default weights from the Stable Diffusion Image-to-Image architecture’s variational autoencoder. This approach required no additional training or fine-tuning beyond these existing models.

### RoentMod development: parameter and prompt selection

The Stable Diffusion Image-to-Image architecture uses two main hyperparameters to control the output image: guidance scale and strength. Higher guidance controls better adherence to the text prompt, often at the expense of lower image quality. Strength controls the extent to which the model can alter the reference image, with 0 meaning no change and 1 meaning maximal change (through additional added noise prior to denoising). We optimized the guidance scale and strength across tested conditions using five randomly selected images with no pathologic findings. We generated all tested conditions for all combinations of ten values for the guidance scale, evenly spaced between 1.5 and 10, and ten values for strength, evenly spaced between 2 and 1 (Supplementary Fig. 7). We had a board-certified radiologist review and notate which parameter combinations produced the most realistic images that contained the requested prompt. From this process, we selected RoentMod’s guidance scale and strength parameters to be 0.4 and 4, respectively, for all experiments in this work.

### Evaluating RoentMod: synthetic evaluation cohort

To evaluate RoentMod’s performance, we first generated a set of synthetic scans by modifying 100 randomly selected scans with no documented pathologic finding from MIMIC-CXR (see Datasets). Per real CXR based on our prompt selection work from before, we had RoentMod produce a synthetic CXR for each of the eight pathology-generating prompts (Supplementary Table 5; 800 synthetic CXRs in total).

Using this synthetic dataset, we tested whether RoentMod can correctly modify existing CXRs with realistic new pathology and preserve unrelated anatomical features of the input individual. We measured RoentMod’s performance along three main metrics: (1) realism and adherence to the text prompt, (2) limited addition of unrelated conditions, and (3) subject-identity preservation.

### Evaluating RoentMod: realism and adherence to the text prompt

We first assessed RoentMod’s ability to realistically produce the prompted pathology when modifying scans. Two board-certified radiologists read all synthetic scans for 100 randomly selected MIMIC-CXR patients in the synthetic cohort for a total of 800 scans read (400 randomly assigned scans per person). We asked each radiologist (Supplementary Table 4) to label presence/absence/unsure of each of the eight findings investigated in the study for each image. We also allowed radiologists to write open-ended notes in reference to image realism and adding extra anomalies. We then manually converted these notes into binary scores for artificialness (1 if artificial) and extra anomaly addition (1 if extra additions present). To explore the effect of intermixing real and synthetic radiographs on reader realism scores and to determine inter-reader reliability, we conducted a second study. We included 50 scans unique to a single reader (25 real, 25 synthetic) and 26 scans read by both readers (13 real and 13 synthetic) for each of eight pathologies (total of 1008 scans—100 × 8 + 26 × 8). Real scans were randomly selected among MIMIC-CXR scans with the desired pathology. We then used the overlapping cases to calculate a real and synthetic Cohen’s κ for each pathology (Supplementary Table 1).

We then assessed the co-occurrence between the radiologist-read labels and the labels according to the specified text prompt to RoentMod (Supplementary Fig. 1). In this matrix, each cell represents the fraction of scans that have the prompted pathology specified in the rows and the radiologist-labeled pathology specified in the columns. Thus, the diagonal of the co-occurrence matrix represents adherence to the text prompt.

### Evaluating RoentMod: limiting unrelated conditions

We then measured how often RoentMod produces unprompted pathology. Using the same co-occurrence matrix (see Methods, Adherence to the text prompt), we assessed the fraction of scans where we prompt RoentMod to produce one condition, but radiologists found another condition on the synthetic scan (i.e., off-diagonal elements of the matrix). We indexed the co-occurrence of RoentMod-generated scans with that of the full real NIH CXR-14 cohort as a surrogate for true co-occurrence.

### Evaluating RoentMod: subject-identity preservation

Finally, we evaluated whether RoentMod-modified scans are anatomically similar to the real input scans using pairwise Fréchet Inception Distance (FID)^32^ across the InceptionV3^33^, XResNet^34^, and CLIP^35^ image embeddings. FID traditionally measures the digitally embedded similarity between two sets of images in the InceptionV3^33^ image embedding space, however, here we only include one image in each set to find the similarity directly between pairs of images and permit evaluation using different embeddings (we call pFID). This work uses pFID to evaluate image similarity between real scans with no pathologic finding paired with a RoentMod synthetic scan from a different person, real scans with no pathologic finding paired with a RoentMod synthetic scan from the same person, and real scans with no pathologic finding paired with a real follow-up scan for the same person when that individual had naturally developed the pathology under investigation within 2 years of the first scan.

To generate paired baseline and follow-up similarity scores, we identified pairs across the entire NIH dataset where these follow-up events occurred, giving us 3261 evaluated pairs across all categories over 2986 total individuals. For patients who had multiple eligible pairs for a given condition, we randomly selected one pair to represent that individual. We list the number of evaluated pairs per condition along with FID results in the appendix (Supplementary Table 2). We then assessed similarity between real and synthetic scans and similarity between two real scans from different individuals using all 2986 of these individuals. We generated synthetic scans of each of our six evaluated pathologies per person, for a total of 2986 pairs of scans per pathology.

### Using RoentMod to evaluate existing image interpretation models

Existing multitask CXR diagnostic models aim to read an input CXR and output one probability score per pathology reflecting the model’s belief that the CXR contains the pathology. We used RoentMod to “stress-test” these diagnostic models. For each real CXR with no pathologic finding, we generated a counterfactual CXR with one added pathology. This allows us to directly measure whether models’ predictions are impacted by off-target pathology. Based on our evaluation of RoentMod, we determined that synthetic scans were of sufficient quality to evaluate the impact of six pathologies: cardiomegaly, edema, pleural effusion, pneumonia, hernia, and pulmonary mass.

### Existing model evaluation: pretrained models

We interrogated four multitask pretrained CXR diagnostic models where model weights were publicly available in this work: two densenet121^65^ architecture models from the TorchXRayVision^64^ library, each trained on different cohorts (TorchXRayVision trained on all cohorts called TorchXRayVision (All), TorchXRayVision trained on the NIH CXR-14 cohort only called TorchXRayVision (NIH)), the ElixrB CXR foundation model^39^, and the Ark+ CXR foundation model^19^. Supplementary Table 3 describes each model’s training datasets, architecture, and diseases that the model can predict.

### Existing model evaluation: quantifying interpretation model shortcuts

We measured the change in predicted probability for each condition of interest by comparing outputs on pairs of (i) real CXR with no pathologic finding from MIMIC-CXR or NIH CXR-14 with (ii) synthetic CXR with synthetically added pathology. Based on the results of the reader study, we removed all synthetic radiographs that were labeled as unrealistic or had an unintended pathology added outside of the eight studied. To control for variable calibration of the tested models, we then converted each output probability to a percentile score (between 0–100%) reflecting how the probability compares to the predictions of the model on the entire cohort. Finally, we calculated the median change in percentile score between the baseline scan and after a pathology was counterfactually added to the scan (Fig. 2 for MIMIC-CXR baselines, Supplementary Fig. 5 for NIH CXR-14 baselines). We separately report the median predicted probability on the baseline and modified scans compared to the true pathology co-occurrence from the radiologist reads (see “Methods” “Evaluating RoentMod adherence to the text prompt”) as a reference standard (Supplementary Fig. 8). To test whether shortcuts were due to the synthetically added pathology, we used saliency maps to highlight the region of each image most driving the prediction of each model. We extracted the ten counterfactual image pairs with the highest change in prediction on the scored pathology and the ten with the lowest change in prediction. We calculated saliency maps before and after the synthetic pathology was added to produce a single heatmap capturing the “change in focus.” We then summarized these heatmaps across each possible added and predicted pathology (6 added pathologies × 6 predicted probability scores = 36 total maps) by taking a pixel-wise average (Supplementary Fig. 3).

### Using RoentMod as data augmentation to improve interpretation models

Lastly, we investigated if we could use RoentMod scans as data augmentation to reduce reliance on shortcuts and improve overall performance of multi-task CXR interpretation models.

### New model: synthetic training cohort

To train our own classifier and avoid data leakage in the process, we generated 10,000 images with no pathologic finding using RoentGen’s^38^ CXR generator (“no acute cardiopulmonary process”). We then had RoentMod produce two synthetic CXRs for each of the original eight pathology-generating prompts (Supplementary Table 5). Overall, RoentMod produced a total of 160,000 synthetic scans for this cohort based on the 10,000 RoentGen baselines for a total of 170,000 synthetic scans.

### New model: model training

We first experimented with single-task or multi-task, the ratio of real to synthetic scans during training, and how off-target pathology should be labeled on counterfactual CXRs. We selected our baseline parameters through a series of small-scale training experiments on 2000 and then 5000 synthetic patients. We trained Densenet121 models to predict each pathology in single- and multi-task setups. Since it is not clear whether RoentMod synthetic scans contain off-target pathology (e.g., prompted cardiomegaly often contains edema), we experimented with different labeling schemes for pathology not mentioned in the text prompt including (i) assuming off-target pathologies were not present, (ii) ignoring off-target pathologies, or (iii) using target values equal to the radiologist-read co-occurrence values (Supplementary Fig. 1). We generated baseline patients with no pathologic finding using RoentGen’s^38^ CXR generator (“no acute cardiopulmonary process”) and then used RoentMod to generate counterfactual scans using the same process as generating the synthetic cohort (see Synthetic Training Cohort) but for the 6 final pathologies, creating a total of seven scans per patient (one no finding, six with findings—one per pathology). After training, we then evaluated each of these small models across the full NIH CXR-14 cohort to produce AUC scores per model per pathology (Supplementary Fig. 9).

From this work, we chose to train a multi-task model on a randomly selected set of 20,000 patients from the NIH CXR-14 cohort (65315 scans) combined with the training portion of the synthetic training cohort of 136,000 scans. All remaining scans from NIH CXR-14 were reserved for testing. We used co-occurrence values from the RoentMod radiologist reads (Supplementary Fig. 1) as the ground truth for off-target pathology for RoentMod synthetic scans (e.g., to label whether pleural effusion is present on a scan that we prompted to add cardiomegaly). We trained the model with a learning rate of 0.0001 for 100 epochs, with a batch size of 32 and early stopping enabled after 50 epochs without improvement.

### New model: evaluation of models trained with RoentMod data augmentation

We then assessed whether models trained with RoentMod augmented data had (i) reduced sensitivity to off-target pathology and (ii) had improved in-distribution and out-of-distribution AUC. We evaluated whether the trained model with RoentMod augmented data was sensitive to the addition of off-target pathology on the full NIH CXR-14 and MIMIC-CXR cohorts for their available labels using the same process as for the pretrained models (Supplementary Table 3). Lastly, we calculated the AUC of our model and all tested pretrained models across held-out NIH CXR-14 participants, the full MIMIC-CXR dataset, and the full PadChest dataset to assess whether data augmentation with RoentMod improves performance in in-distribution, in the generator’s training distribution, and in out-of-distribution datasets, respectively.

### Ethics information

This study was conducted in accordance with the Declaration of Helsinki under supervision by the Massachusetts General Brigham IRB. This study was conducted under a waiver of informed consent for secondary analysis of deidentified data.

## Supplementary information

Supplementary Revision.

## Data Availability

Pre-existing cohorts (MIMIC-CXR, NIH-CXR 14, and PadChest) are publicly available after completing each cohort’s access requirements. MIMIC-CXR requires users to complete a human subjects research training and request access. NIH CXR-14 is publicly available through Kaggle or the NIH website without any additional training or credentials, and PadChest requires submitting a formal data request. Our synthetic cohort will be made available for research by request upon publication.

## Code availability

All models and code will be available upon publication.
