# Supplementary material for: RoentMod: a synthetic chest X-ray modification model to identify and correct image interpretation model shortcuts
Source: NPJ Digit Med. 2026 Mar 6;9:324. doi: 10.1038/s41746-026-02497-6 (PMC13087218; doi:10.1038/s41746-026-02497-6)
Supplement: Supplementary file 1 — Supplementary Revision. [file 41746_2026_2497_MOESM1_ESM.pdf]

Supplemental Material

Supplemental Figure 1: Co-occurrence between prompted pathology and radiologist read pathology in RoentMod generated counterfactual CXRs (right) vs. observed co-occurrence between pathologies in NIH CXR-14 (left)	2
Supplemental Figure 2: Comparison between radiologist-evaluated reads (columns) vs. RoentMod-prompted pathology (rows) stratified by reader	3
Supplemental Table 1: Cohen's κ for overlapping reads stratified by pathology and real vs. synthetic instance.	4
Supplemental Table 2: Fréchet Inception distance between unpaired synthetic-real scans (control score), paired synthetic-real scans (model score), and real scans with real follow-up scans (real score) across embeddings (rows) and conditions (columns)	5
Supplemental Figure 3: Pixel intensity change on RoentMod added pathology in MIMIC-CXR.	6
Supplemental Table 3: Publicly available chest radiograph interpretation models evaluated in this study	7
Supplemental Figure 4: Change in saliency heatmaps on TorchXRayVision (NIH) between MIMIC-CXR and RoentMod scan pairs.	8
Supplemental Figure 5: Effect of adding pathologies on predicted probabilities from existing multitask CXR interpretation models (**a**) and our RoentMod-trained multitask CXR interpretation model (**b**) on NIH CXR-14 synthetic scans.	9
Supplemental Figure 6. Co-occurrence of findings in NIH, MIMIC-CXR, CheXpert, and PadChest cohorts	10
Supplemental Table 4. Characteristics of NIH CXR-14, MIMIC-CXR, CheXpert, and PadChest cohorts	11
Supplemental Figure 7: Effect of strength and guidance on RoentMod-generated chest radiographs	12
Supplemental Table 5: RoentMod Prompts (original and pruned) and reader instructions	13
Supplemental Figure 8: Change in predicted probability of six conditions from baseline scans with no pathology to counterfactual scans with added pathology	14
Supplemental Figure 9: Initial disease prediction to determine model training parameters	15

Supplemental Figures and Tables

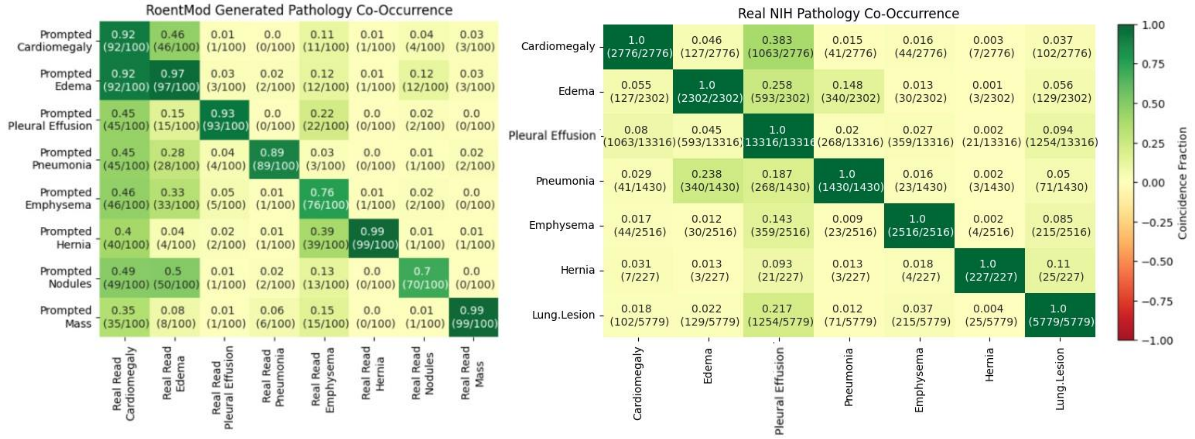

Supplemental Figure 1: Co-occurrence between prompted pathology and radiologist read pathology in RoentMod generated counterfactual CXRs (right) vs. observed co-occurrence between pathologies in NIH CXR-14 (left)

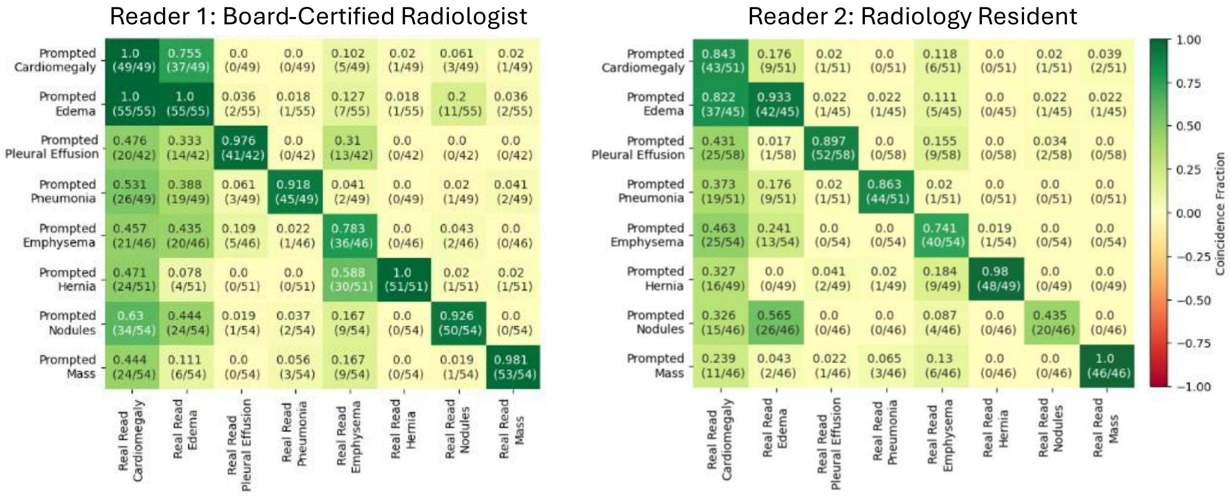

Supplemental Figure 2: Comparison between radiologist-evaluated reads (columns) vs. RoentMod-prompted pathology (rows) stratified by reader

	Cardiomegaly	Edema	Emphysema	Hernia	Mass	Pneumonia	Nodules	Pleural Effusion
**Real Scans**	0.55 (0.09)	0.31 (0.14)	0.17 (0.21)	0.78 (0.12)	0.56 (0.25)	0 (0.70)	1 (0)	0.75 (0.08)
**Synthetic Scans**	0.48 (0.11)	0.19 (0.09)	0.30 (0.10)	1 (0)	0.96 (0.04)	0.67 (0.13)	0.64 (0.10)	0.63 (0.10)

Supplemental Table 1: Cohen's κ for overlapping reads stratified by pathology and real vs. synthetic instance

FID Median (IQR) (N=2986)		Cardiomegaly N=395	Edema N=167	Pneumonia N=302	Pleural Effusion N=1764	Hernia N=38	Pulmonary Mass N=897
**InceptionV3 Embeddings**	**Control Score**	211.05 (73.8)	222.99 (75.9)	205.12 (73.0)	202.96 (71.2)	223.97 (74.3)	202.79 (72.2)
	**Real Score** (≤ 2 yrs follow up)	**138.09 (66.78)**	**143.85 (65.9)**	**138.81 (60.1)**	**141.55 (71.9)**	**125.44 (51.9)**	**133.30 (72.0)**
	**Model Score**	156.53 (60.48)	184.68 (69.2)	147.70 (61.5)	156.73 (63.5)	182.52 (62.3)	159.68 (64.0)
**XResNet Embeddings**	**Control Score**	196.05 (80.66)	195.42 (78.3)	190.30 (81.9)	182.67 (74.3)	201.12 (76.5)	190.21 (74.3)
	**Real Score** (≤ 2 yrs follow up)	119.07 (63.68)	**129.10 (69.5)**	**114.31 (59.8)**	121.24 (63.2)	**116.90 (53.9)**	**113.21 (66.4)**
	**Model Score**	**118.99 (52.93)**	134.35 (57.6)	114.80 (61.5)	**121.01 (51.9)**	147.04 (57.1)	123.31 (52.7)
**CLIP Embeddings**	**Control Score**	0.1138 (0.184)	0.1094 (0.175)	0.1161 (0.196)	0.1168 (0.196)	0.1101 (0.177)	0.1207 (0.200)
	**Real Score** (≤ 2 yrs follow up)	0.0825 (0.169)	0.0608 (0.108)	0.0797 (0.214)	0.0726 (0.170)	0.0848 (0.201)	0.0747 (0.175)
	**Model Score**	**0.0091 (0.158)**	**0.0149 (0.022)**	**0.0077 (0.014)**	**0.0094 (0.019)**	**0.0150 (0.025)**	**0.0108 (0.196)**

Supplemental Table 2: Fréchet Inception distance between unpaired synthetic-real scans (control score), paired synthetic-real scans (model score), and real scans with real follow-up scans (real score) across embeddings (rows) and conditions (columns)

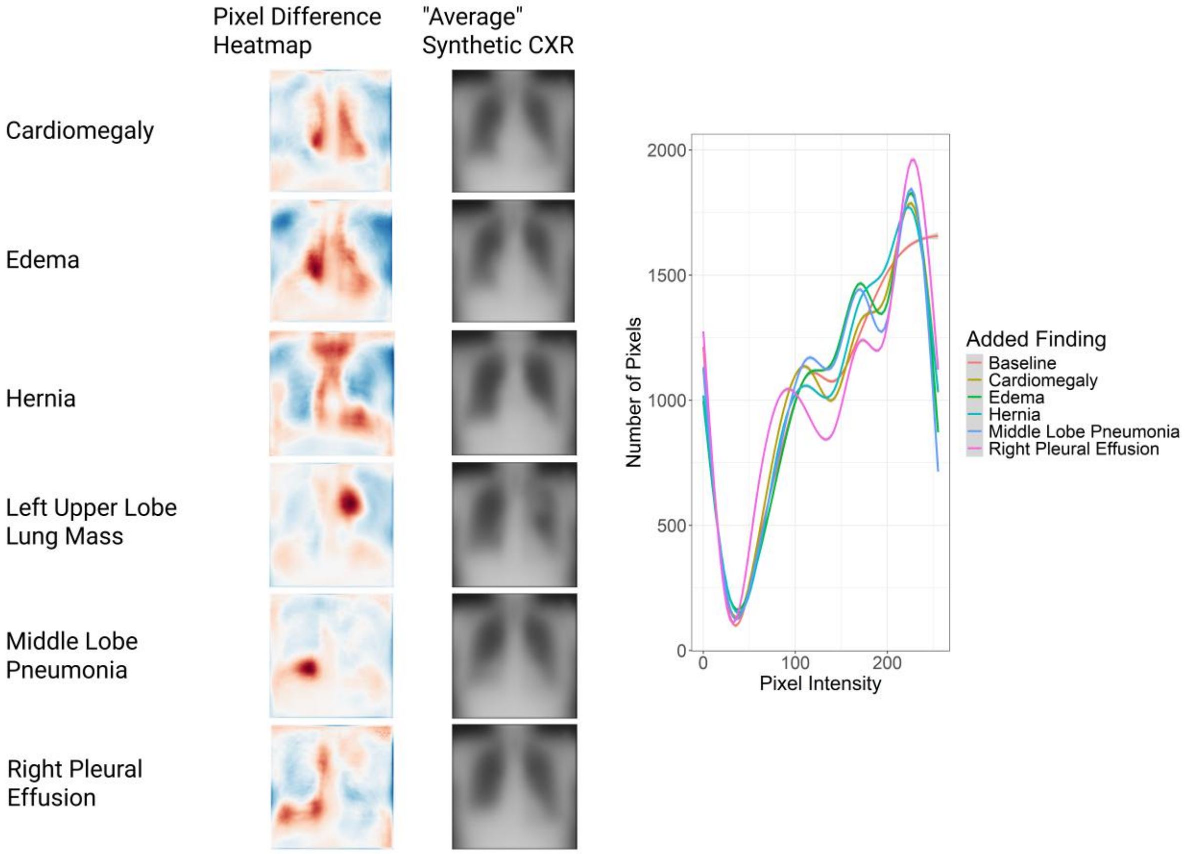

Supplemental Figure 3: Assessment of subtle image changes induced by RoentMod editing. Changes in spatially resolved pixel intensity (left), average pixel intensity (middle), and distribution of pixel intensities (right) in radiographs from the MIMIC-CXR reader study cohort modified to add pathology by RoentMod.

Model Name	Model Type	Training Datasets	Model Architecture	Tested Model Predictions	Code Reference
txrv-all	Multitask classification	NIH CXR-14, MIMIC-CXR, CheXpert, PadChest	Densenet121<br>224px input image resolution	Cardiomegaly, Edema, Pleural Effusion, Mass, Pneumonia, Hernia	https://github.com/mlmed/torchxrayvision?tab=readme-ov-file
txrv-nih	Multitask classification	NIH CXR-14	Densenet121<br>224px input image resolution	Cardiomegaly, Edema, Pleural Effusion, Mass, Pneumonia, Hernia	https://github.com/mlmed/torchxrayvision?tab=readme-ov-file
ElixrB (Google)	Foundation	MIMIC-CXR, 485,082 proprietary CXRs from India, 165,182 proprietary CXRs from a hospital in Illinois, USA	EfficientNet-L2 CNN image encoder and a BERT-based text encoder, trained jointly via supervised contrastive, CLIP, and BLIP-2 losses<br>1024px input image resolution	Cardiomegaly, Edema, Pleural Effusion, Mass, Pneumonia, Hernia	https://huggingface.co/google/cxr-foundation
Ark+	Foundation	MIMIC-CXR, NIH CXR-14, CheXpert, RSNA Pneumonia, VinDr-CXR, Shenzhen	Swin-Transformer-based image encoder with a momentum teacher-student architecture and projection head<br>1024px input image resolution	Cardiomegaly, Edema, Pleural Effusion, Mass, Pneumonia, Hernia	https://github.com/jlianglab/Ark

Supplemental Table 3: Publicly available chest radiograph interpretation models evaluated in this study

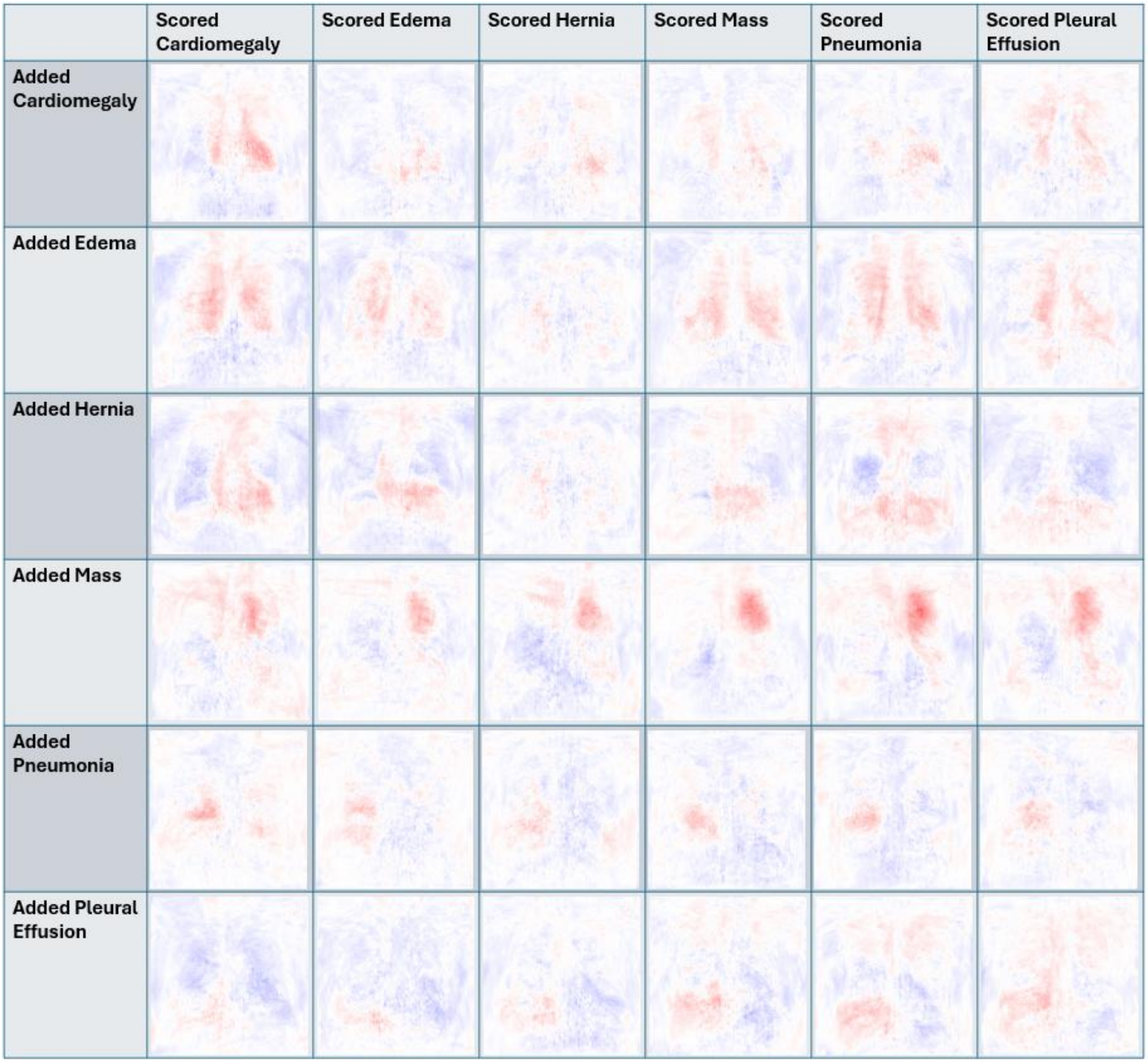

Supplemental Figure 4: Change in saliency heatmaps on TorchXRayVision (NIH) between MIMIC-CXR and RoentMod scan pairs. Per represented pathology-added and pathology-scored combination, red portions represent an increased activation for the scored condition in the RoentMod scan in those pixels on average, while blue portions represent a decreased activation in the RoentMod scan.

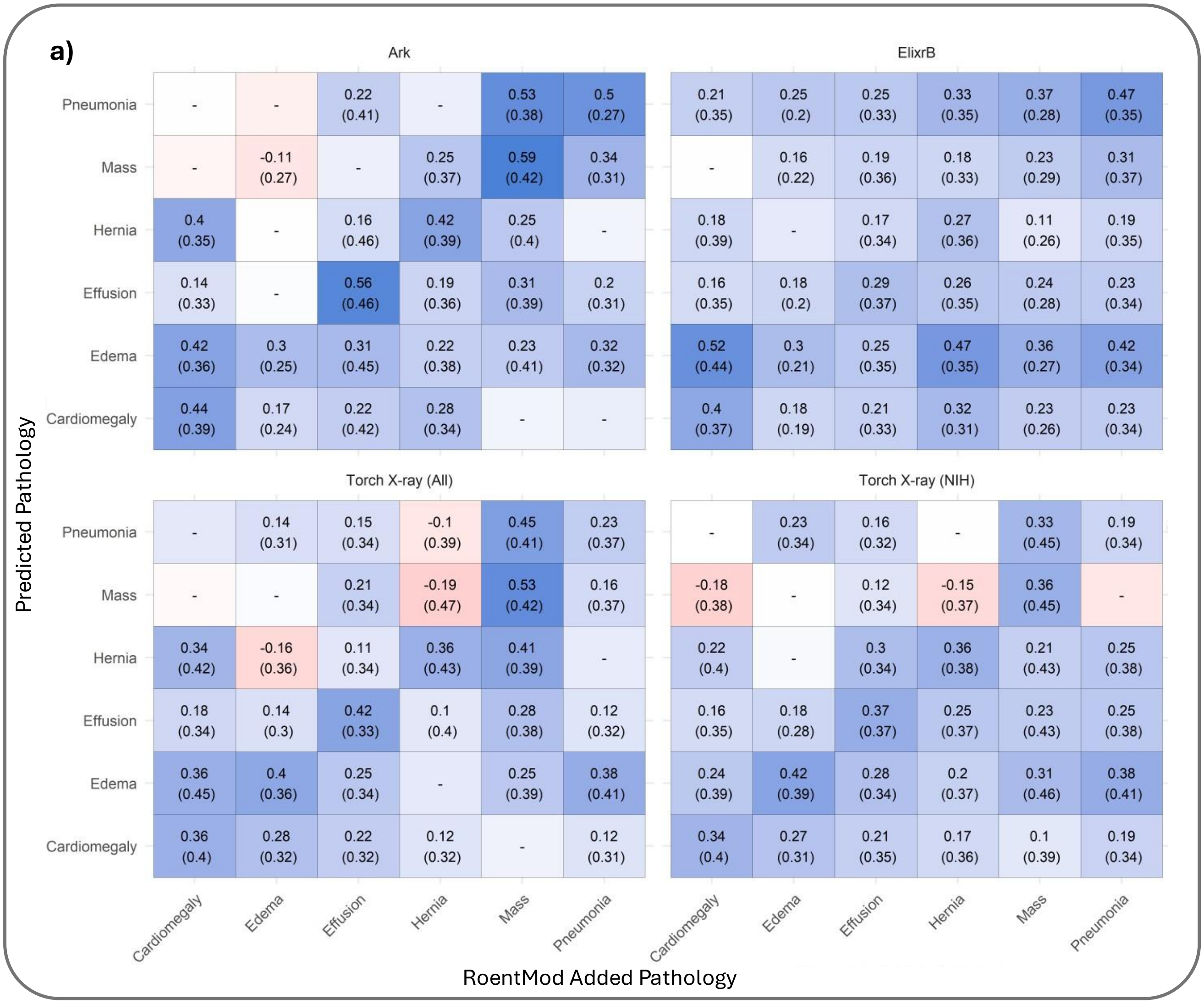

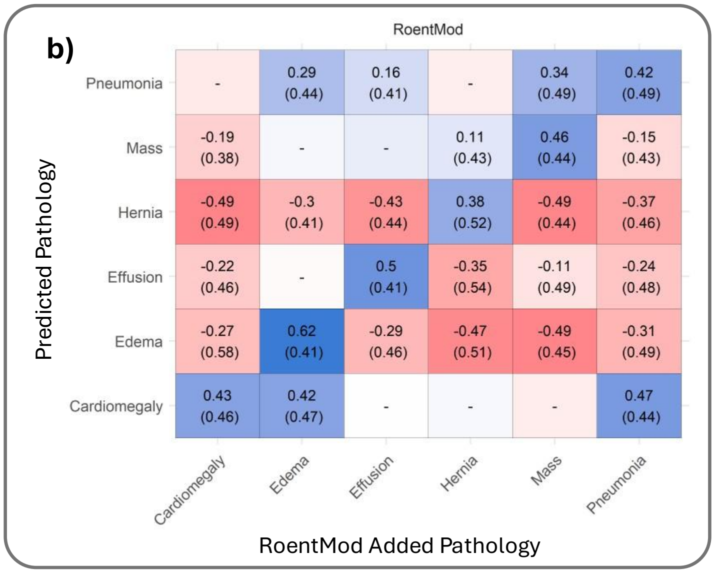

Supplemental Figure 5: Effect of adding pathologies on predicted probabilities from existing multitask CXR interpretation models (**a**) and our RoentMod-trained multitask CXR interpretation model (**b**) on NIH CXR-14 synthetic scans. Blue boxes indicate greater sensitivity to adding pathology. Blank boxes indicate no change between baseline and RoentMod-generated counterfactuals. Values reflect the median change in predicted probability percentile after pathology is added to scans with no finding in NIH CXR - 14. So, the value 0.29 (0.44) in **b** in the pneumonia row and edema column represents that median pneumonia predictions increased 0.29 percentile, or rank within the distribution, when RoentMod added Edema with an interquartile range of 0.44 percentile. We refer to the TorchXRayVision model trained on all cohorts as TorchXRayVision (all) and the TorchXRayVision model trained on the NIH CXR-14 cohort only as TorchXRayVision (NIH).

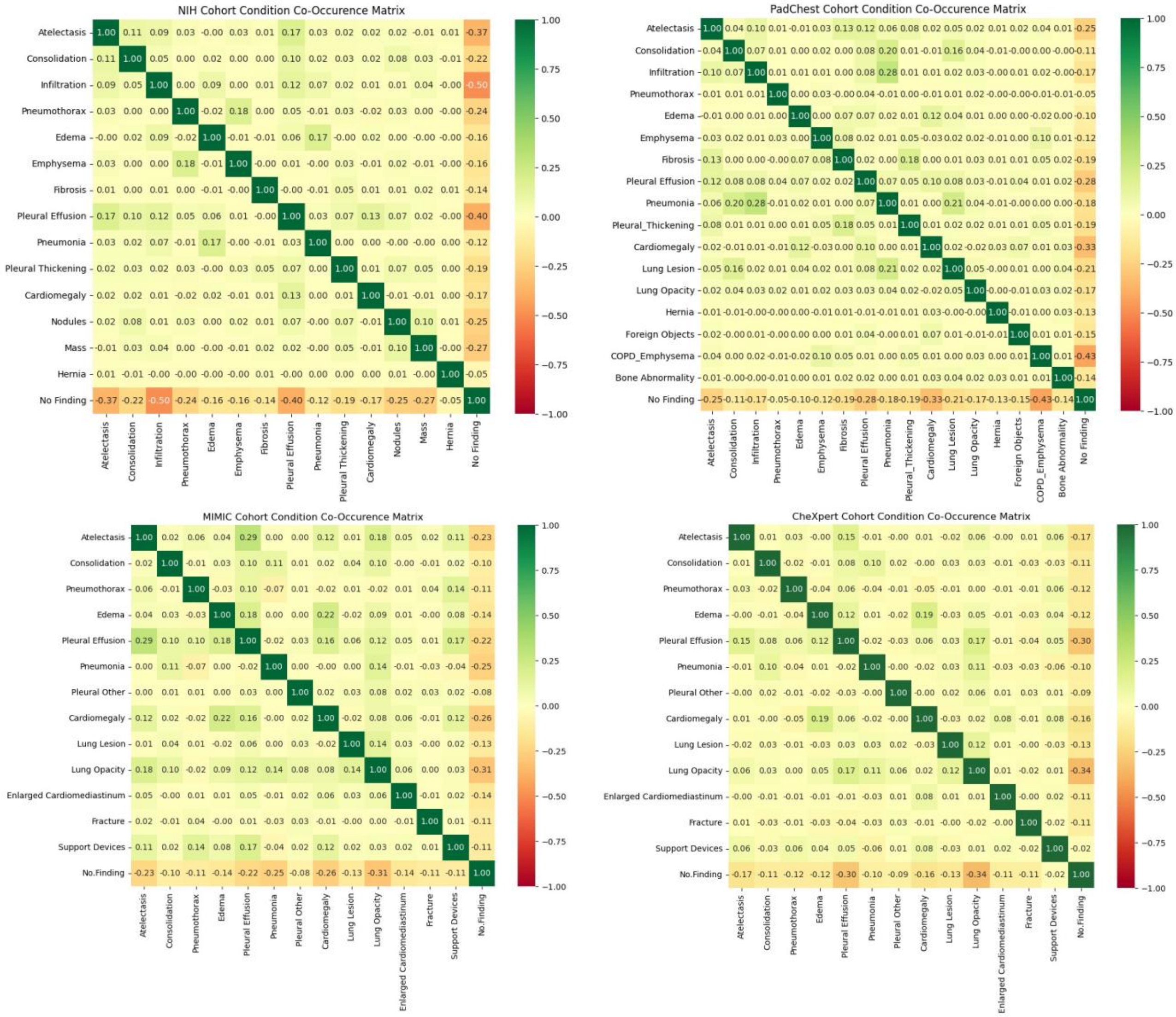

Supplemental Figure 6: Co-occurrence of findings in NIH CXR-14, MIMIC-CXR, CheXpert, and PadChest cohorts

	**NIH CXR-14** (64628 scans) N = 27713	**MIMIC-CXR** (94067 scans) N = 44642	**CheXpert** (29453 scans) N = 20574	**PadChest** (88109 scans) N = 59085
Mean Age (SD)	47.8 (15.0)	56.1 (19.0)	57.1 (17.7)	58.6 (17.4)
% Female (N female)	46% (12759)	53.0% (23679)	38% (7805)	52.4% (30982)
Atelectasis % (# scans)	8.7% (5614)	16.9% (15912)	10.9% (3199)	5.9% (5199)
Consolidation % (# scans)	2.3% (1463)	4.0% (3745)	5.1% (1498)	1.2% (1072)
Infiltration % (# scans)	13.9% (8976)	--	--	2.9% (2563)
Pneumothorax % (# scans)	5.1% (3268)	4.5% (4220)	6.1% (1804)	0.2% (207)
**Edema % (# scans)**	0.4% (268)	7.1% (6671)	5.8% (1710)	0.9% (791)
**Emphysema % (# scans)**	2.3% (1473)	--	--	1.3% (1187)
Fibrosis % (# scans)	2.2% (1394)	--	--	3.3% (2935)
**Pleural Effusion % (# scans)**	10.0% (6450)	16.1% (15152)	27.4% (8081)	7.1% (6240)
**Pneumonia % (# scans)**	0.9% (586)	19.8% (18589)	4.1% (1198)	3.2% (2789)
Pleural Thickening % (# scans)	3.7% (2366)	--	--	3.6% (3213)
Pleural Other % (# scans)	--	2.5% (2390)	3.4% (1012)	0.5% (433)
**Cardiomegaly % (# scans)**	2.4% (1520)	21.1% (19844)	9.9% (2910)	9.9% (8680)
**Lung Lesion % (# scans)**	5.4% (3466)	6.0% (5670)	7.2% (2122)	4.2% (3705)
Lung Opacity % (# scans)	6.4% (4106)	26.9% (25300)	33.1% (9744)	2.9% (2538)
Enlarged Cardiomediastinum % (# scans)	--	7.2% (6758)	4.9% (1438)	0.7% (631)
Fracture % (# scans)	--	4.5% (4262)	4.8% (1411)	4.3% (3762)
**Hernia % (# scans)**	0.3% (191)	--	--	1.7% (1481)
Support Devices % (# scans)	--	16.7% (15738)	27.7% (8146)	4.5% (3944)
**No Finding % (# scans)**	58.0% (37452)	20.8% (19561)	18.7% (5513)	50.2% (44259)

Supplemental Table 4: Characteristics of NIH CXR 14, MIMIC-CXR, CheXpert, and PadChest cohorts

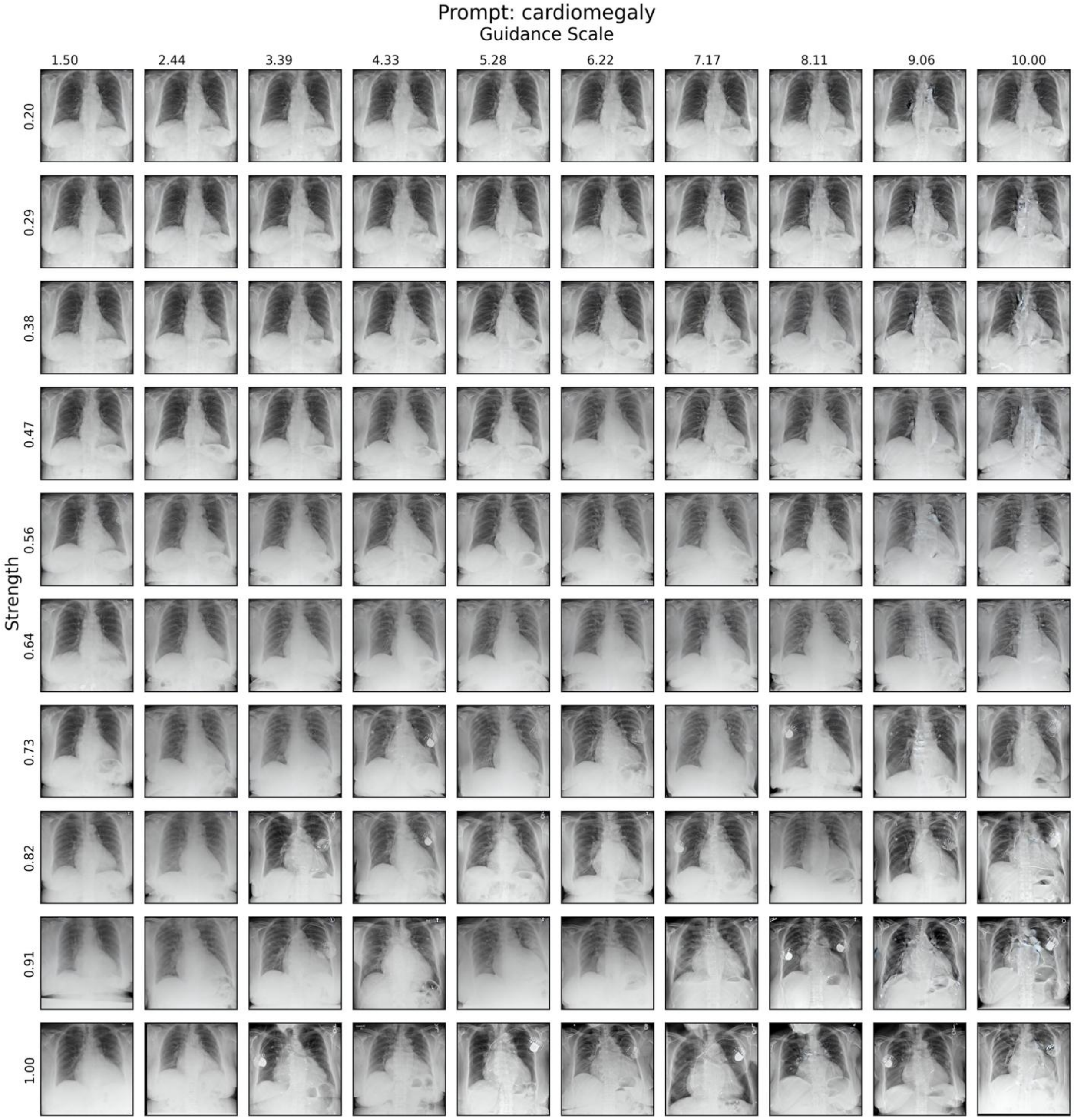

Supplemental Figure 7: Effect of strength and guidance on RoentMod-generated chest radiographs

Target Pathology	Tested Prompts	Final Prompts
No finding	“no acute cardiopulmonary process”	“no acute cardiopulmonary process”
Cardiomegaly	“cardiomegaly”	“cardiomegaly”
Edema	"edema", "butterfly edema"	“edema”
Pneumonia	"pneumonia", "right upper lobe pneumonia", "left upper lobe pneumonia", "middle lobe pneumonia", "right lower lobe pneumonia", "left lower lobe pneumonia"	“middle lobe pneumonia”
Pleural Effusion	“right pleural effusion”, "left pleural effusion", "right pleural effusion"	“right pleural effusion”
Emphysema	"emphysema", "severe emphysema", "panlobular emphysema"	--
Hernia	“hernia”	“hernia”
Pulmonary Nodules	"solitary lung nodule", "multiple pulmonary nodules"	--
Pulmonary Mass	"right upper lobe mass", "left upper lobe mass", "middle lobe mass", "right lower lobe mass", "left lower lobe mass"	“left upper lobe mass”

Reader Instructions
For each of the following anonymized scans in your assigned set, please locate the scan number corresponding to the scan ID c olumn (ie. row with scan ID 9 for scan 9.jpg). For each row, fill out each column to indicate if the specified condition is present (1), not pres ent (0 or blank), or unsure (2). (ie. if scan x has Cardiomegaly and nothing else, put a 1 in the cardiomegaly column for that row and nothing everywhere else) I f anything about the particular image is odd or you need to note something beyond these labels, please type that information into the corresponding row's Notes col umn.

Supplemental Table 5: RoentMod Prompts (original and pruned) and reader instructions

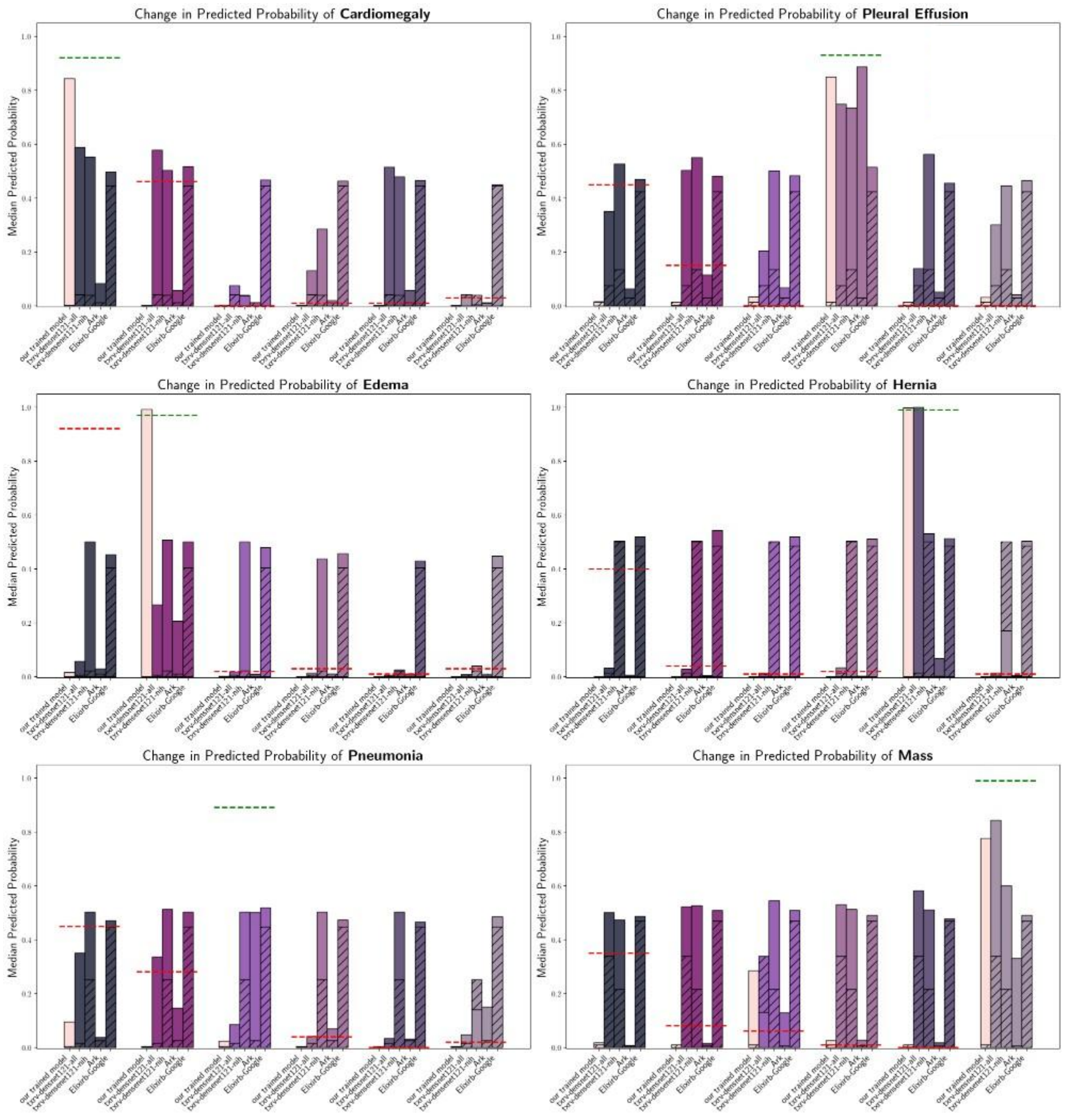

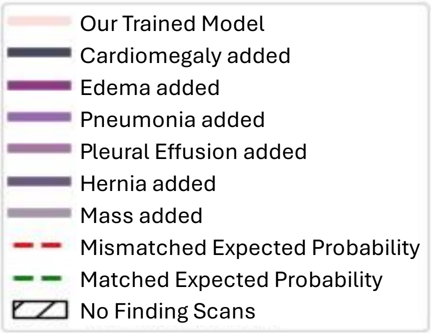

Supplemental Figure 8: Change in predicted probability of six conditions from baseline scans with no pathology to counterfactual scans with added pathology

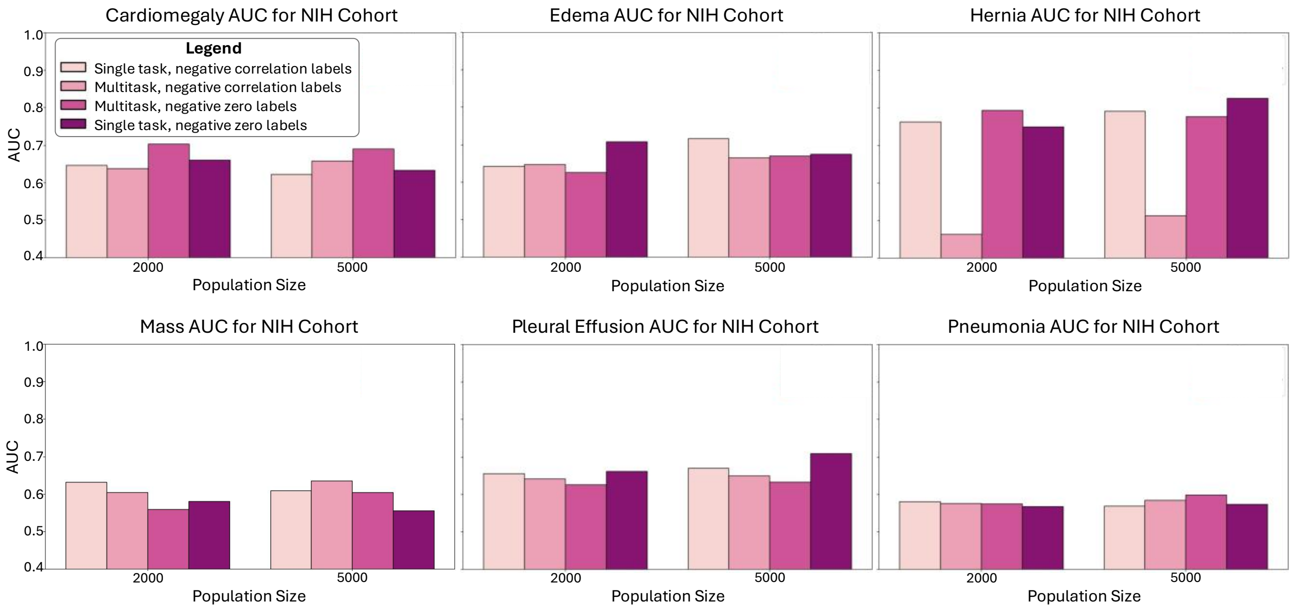

Supplemental Figure 9: Small-scale experiments to determine at scale diagnostic model training parameters across training set size (x-axis), correlation label scheme, and multitask vs. single task model design. Because RoentMod can add co-occurring pathologies in addition to the requested disease (Supplemental Figure 2), we tested labeling unrequested pathologies as their co-occurrence probability (“negative correlation labels”) versus assuming those pathologies are not present (“negative zero labels”).
